# Huai Qi Huang corrects the balance of Th1/Th2 and Treg/Th17 in an ovalbumin-induced asthma mouse model

**DOI:** 10.1042/BSR20171071

**Published:** 2017-12-22

**Authors:** Peng Liang, Shao Peng, Man Zhang, Yingying Ma, Xinggang Zhen, Huijuan Li

**Affiliations:** 1 Department of Pediatrics, The First Affiliated Hospital of Zhengzhou University, Zhengzhou 450052, China; 2 Department of Respiration, Children’s Hospital of Zhengzhou City, Zhengzhou 450053, China

**Keywords:** asthma, Huai Qi Huang, Th1/Th2, Treg/Th17

## Abstract

The present study is designed to determine whether Huai Qi Huang has immunoregulatory effects on the (helper T (Th)) Th1/Th2 and regulatory T cell (Treg)/Th17 balance in ovalbumin (OVA)-induced asthma model mice. Asthma model mice were constructed by OVA treatment and Huai Qi Huang was administered. The amount of migrated inflammatory cells in the bronchoalveolar lavage fluid (BALF) from the OVA mice was counted. The total IgE in the sera was detected by the IgE ELISA kit. Cell suspensions from the lung were stained with antibodies specific for CD4 and the master transcription factors for Th1 (T-box expressed in T cells (T-bet)), Th2 (GATA-binding protein 3 (Gata-3)), Th17 (retinoic acid related orphan receptor γt (RORγt)), and Treg (forkhead box p3 (Foxp3)). The left lobe of the lung was used to prepare a single-cell suspension for flow cytometry to determine whether Huai Qi Huang influenced CD4^+^ T-cell subsets. Histological analyses were performed by using Hematoxylin and Eosin staining. The mRNA expression levels of the transcription factors were detected by using qRT-PCR. Huai Qi Huang inhibited infiltration of inflammatory cells into the lung, reduced influx of eosinophils (EOSs), lymphocytes (LYMs), neutrophils (NEUs), and macrophages (MACs) in the BALF, and decreased IgE in the serum in OVA-treated mice. Huai Qi Huang could regulate Th1/Th2 and Treg/Th17 via the re-balance of cytokine profiles and change the mRNA expression levels of the transcription factors, T-bet/Gata-3 and Foxp3/RORγt in OVA-treated mice. Our results showed that Huai Qi Huang could correct the imbalance of Th1/Th2 and Treg/Th17 in OVA-induced asthma model mice, indicating its effects on inhibiting the development and severity of asthma.

## Introduction

Asthma is histologically characterized by goblet cell hyperplasia, inflammatory cell infiltration of the bronchial mucosa, thickening of the submucosa, and epithelial cell desquamation [1]. Helper T (Th) cells play a crucial role in dysfunction of immune, which contributes to the progress of asthma [2]. Th cells are divided into four subtypes: interferon (IFN)-γ-secreting Th1, interleukin (IL)-4-secreting Th2, IL-17-producing Th17 cells, and CD4^+^ CD25^+^ Foxp3^+^ regulatory T cells (Tregs) [3]. Th1 cells release IL-2, IL-12, IFN-γ, and tumor necrosis factor (TNF)-β [4]. Cazzola and Polosa [5] indicated that therapies interfering with Th1-derived cytokines may be a considerable option for asthma patients who are particularly resistant to typical treatment modalities. Th2-type cytokines, such as IL-4, IL-5, and IL-13, are reported to drive the accumulation of eosinophils (EOSs) in the lungs of asthmatic patients [6]. Th17 cells could release IL-17 and were reported to recruit neutrophils (NEUs) and attract EOS indirectly, further exacerbating asthma attacks [7,8]. Treg cells exert their effect via cytokines such as IL-10 and TGF-β or through directing cell-to-cell contact, which are major regulators of autoimmunity and also play an important role in asthma [9,10].

Recently, it is well accepted that the imbalance of Th1/Th2 and Treg/Th17 may be key factors that contribute to asthma severity [11]. The production of cytokines by Th2 cells blocks the production of cytokines by Th1 and natural killer cells. In addition, Th1 cells can inhibit the differentiation and proliferation of mastocytes, basophils, and EOSs, whose activities are controlled by the synthesis of cytokines by Th2 cells [4]. Moreover, immunoregulatory therapies that initiate a shift from Th2 to Th1 responses have been also explored in previous study [12]. Besides, the transcription factor Gata-binding protein 3 (Gata-3), which can drive Th2 cell differentiation and control expression of Th2 cytokines, is found suppressed by T-box expressed in T cells (T-bet), a Th1-specific transcription factor [13–15]. Besides, forkhead box p3 (Foxp3), the indispensable master transcription factor for the development and function of Treg, was reported to be associated with and inhibit retinoic acid related orphan receptor γt (RORγt), thus aborting the differentiation of Th17 cells [16]. Importantly, a previous study indicated the imbalance of Th1/Th2 and Treg/Th17 in a mouse asthma model [17].

Huai Qi Huang, a mixture of Chinese herbs, contains *Trametes robiniophila murr* (Huaier), wolfberry fruit, and *Polygonatum* [18]. Huai Qi Huang could prevent podocyte injury, reduce proteinuria, inhibit inflammatory cytokine expression, ameliorate tubulointerstitial damage, and inhibit macrophage (MAC) infiltration in adriamycin nephrotic rats [19]. Importantly, Li et al. [20] indicated that Huai Qi Huang treatment may increase the IFN-γ expression in plasma and bronchoalveolar lavage fluid (BALF) and the phagocytosis of alveolar MAC in asthmatic rats. Huang [21] suggested that Huai Qi Huang particles could be used for recurrent respiratory tract infections by affecting the Th17-mediated NEU inflammation to enhance the body’s immune system in children. These investigations indicated the important role of Huai Qi Huang in the treatment of asthma, and we speculated that Huai Qi Huang may be used for the treatment of asthma by restoration of the balance of Th1/Th2 and Treg/Th17. The present study is focussed on the immunoregulatory effects of Huai Qi Huang on the Th1/Th2 balance in ovalbumin (OVA)-induced asthma model mice.

## Methods

### Animals

Female C57BL/6J mice (6–8 weeks), free of specific pathogens, were obtained from the Shanghai Laboratory Animal Centre, Chinese Academy of Sciences. All experimental animals were maintained under a protocol approved by The First Affiliated Hospital of Zhengzhou University.

### Groups

Mice were randomly divided into four groups (*n*=10 in each group): the control group, the OVA group, OVA mice that were treated with Huai Qi Huang (OVA + Huai Qi Huang group), and OVA mice that were treated with dexamethasone (Dex) (OVA + Dex). Dex is widely used to treat asthma and served as a positive control in the present study. Mice in the last three groups were intraperitoneally (i.p.) injected with 100 μg OVA (Sigma, St. Louis, MO, U.S.A.) emulsified in 1 mg aluminum hydroxide (Pierce Chemical Co., Rockford, IL, U.S.A.) with a total volume of 0.2 ml on days 0–14. One day later, mice were challenged for 30 min via the airway with OVA (5% OVA) by ultrasonic nebulizer each day on days 15–22 consecutively. In the OVA + Huai Qi Huang group, OVA-treated mice were administered with Huai Qi Huang (0.4 g/100 g body weight; Qidong Gaitianli Pharmaceutical Co., Ltd, Zhunzi, B20020074) daily by intragastric gavage. In the OVA + Dex group, OVA-treated mice were administered Dex phosphate (Sigma, 10% solution in PBS) by intragastric gavage for 1 h before OVA aerosol on days 15–22. Mice in the control group received the same schedule for sensitization and were administered with an equivalent amount of 0.9% sterile saline instead of OVA.

### Analysis of BALF samples and sera

For collection of BALF samples and sera, mice were bled by retro-orbital puncture using heparinized capillary tubes as described previously [17]. After extracting the blood samples, tracheas were inserted with a catheter by way of an incisal opening sited in the cervical part, and airway lumina were washed. The pooled BALF was centrifuged and the number of total cells was counted with a hemacytometer. For differential cell counting, smears of BALF cells were stained with Wright’s stain and were counted by two independent blinded investigators. The commercially available ELISA kits (San Diego, CA, U.S.A.) were used to detect the cytokine and chemokine in BALF supernatant. The total IgE in the sera was detected by the IgE ELISA Kit (BioLegend, San Diego, CA, U.S.A.).

### Histopathology analysis

After BALF was obtained, the upper right lung lobe was removed, fixed in 10% neutral buffered formalin for 24 h, and then specimens were dehydrated and embedded in paraffin. Five-micrometer sections of fixed embedded tissues were cut on a Leica model 2165 rotary microtome (Leica, Nussloch, Germany) and stained with Hematoxylin and Eosin. Histological analyses were performed by two independent pathologists blinded to the treatment groups.

### RNA preparation and real time quantitative reverse transcription polymerase chain reaction (qRT-PCR)

The portions of the right lung lobes were used for total RNA extraction by using RNAzol (Invitrogen; Thermo Fisher Scientific, Inc., Waltham, MA, U.S.A.). cDNA was prepared by reverse transcription of ssRNA using the High Capacity cDNA Reverse Transcription Kit (Applied Biosystems; Thermo Fisher Scientific, Inc., MA, U.S.A.), according to the manufacturer’s instructions. qRT-PCR was carried out using the SYBR® Premix Ex Taq™ kit (Takara Bio, Inc., Otsu, Japan), according to the manufacturer’s instructions. The 20 μl reaction mix consisted of 2 μl 30-fold diluted First-strand cDNA, 10 μl 2× SYBR® Premix Ex Taq™, 0.4 μl 10 μM forward and reverse primer, 0.4 μl 50× ROX Reference Dye, and 6.8 μl diethy pyrocarbonate (DEPC)-treated water. The primer pairs used in these reactions were as follows: T-bet, forward, 5′-GCCAGGGAACCGCTTATATG-3′, reverse, 5′-GACGATCATCTGGGTCACATTGT-3′; Gata-3, forward, 5′-GAGGTGGACGTACTTTTTAACATCG-3′, reverse, 5′-GGCATACCTGGCTCCCGT-3′; RORγ, forward, 5′-CCGCTGAGAGGGCTTCAC-3′, reverse, 5′-TGCAGGAGTAGGCCACATTACA-3′; Foxp3, forward, 5′-CCCAGGAAAGACAGCAACCTT-3′, reverse, 5′-TTCTCACAACCAGGCCACTTG-3′; GAPDH, forward, 5′-ACCACAGTCCATGCCATCAC-3′, reverse, 5′-TCCACC ACCCTGTTGCTGTA-3′. Reactions were performed in an ABI7300 Real-Time quantitative instrument (Applied Biosystems; Thermo Fisher Scientific, Inc., MA, U.S.A.). The thermocycling conditions were as follows: initial denaturation at 95°C for 30 s, followed by 40 cycles of 95°C for 5 s, and 60°C for 31 s. The expression level of the internal control glyceraldehyde-3-phosphate dehydrogenase (GAPDH) was used as a housekeeping gene, and the comparative 2^−ΔΔ*C*q^ method [22] was used to quantitate gene expression levels.

### Flow cytometry

The left lobe of the lung was used to prepare a single-cell suspension for flow cytometry as described previously [17]. In brief, the left lobe was digested and a single-cell suspension was obtained, and then stained with fluorescence-labeled antibodies specific for CD4-conjugated APC-eFluor 780 (RM4-5), T-bet-conjugated eFluor 660 (eBio4B10), Gata3-conjugated PerCP-eFluor 710 (TWAJ), Foxp3-conjugated Alexa Fluor 488 (FJK-16s), and RORγt-conjugated PE (AFKJS), or the istotype control. All the antibodies were from eBioscience (San Diego, U.S.A.). The cells were blocked with anti-CD16/32 to reduce non-specific binding and stained with anti-CD4. Subsequently, the cells were fixed, permeabilized, and further stained with the transcription factor antibodies.

### Statistical analysis

Data were expressed as the mean ± S.E.M. Each experiment was repeated at least three times. Statistical analysis was performed with SPSS software (SPSS, Chicago, IL, U.S.A.) by using one-way ANOVA and least significant difference (LSD) tests. *P*<0.05 was considered statistically significant.

## Results

### Huai Qi Huang inhibited infiltration of inflammatory cells into the lung

**Figure 1:**
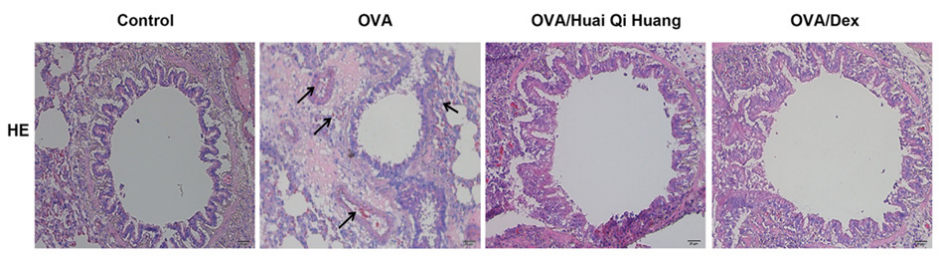
Huai Qi Huang inhibited infiltration of inflammatory cells into the lung The lungs were removed 24 h after the OVA challenge. Sections were stained by Hematoxylin and Eosin (magnification 200×). The experiment was repeated at least six times with similar results and the representative result was shown. *n*=10 in each group. Arrows indicate the infiltration of inflammatory cells. HE, hematoxylin and eosin.

As compared with control mice, the amount of inflammatory cells infiltrating the airway mucosa in the mice that were treated with OVA was increased, tracheal lumen became narrower, smooth muscle and basement membrane were thicker. Importantly, Huai Qi Huang significantly inhibited the infiltration of EOSs and NEUs into the lungs in the OVA-treated mice. Mice treated with Dex also showed marked reductions in the infiltration of EOSs in the peribronchiolar and perivascular regions (Figure 1).

### Huai Qi Huang reduced influx of EOSs, lymphocytes, NEUs, and MACs in the BALF

**Figure 2:**
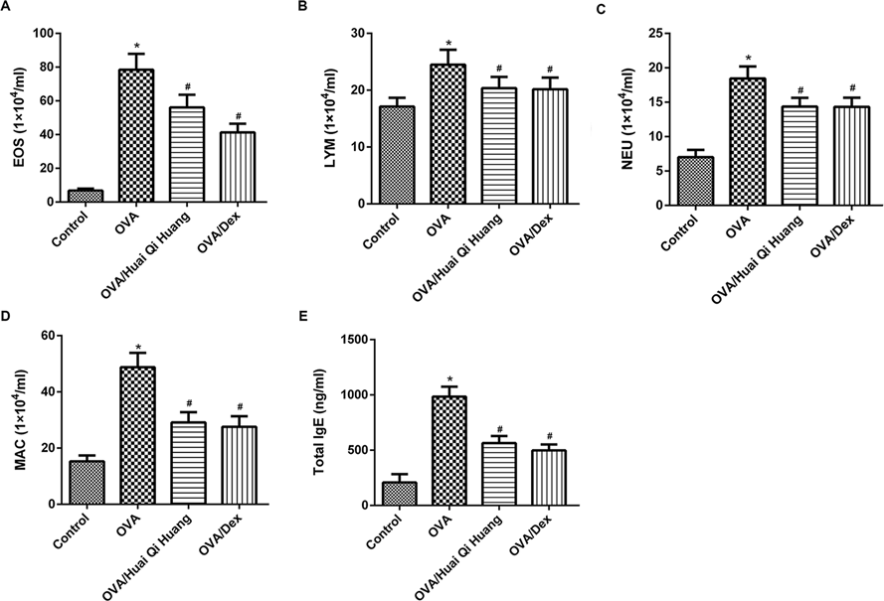
Huai Qi Huang reduced influx of EOSs, LYMs, NEUs, and MACs in the BALF Administration of Huai Qi Huang or Dex significantly reduced the amount of migrated inflammatory cells: EOSs (**A**), LYMs (**B**), NEUs (**C**), and MACs (**D**) in the BALF from the OVA mice. (**E**) Administration of Huai Qi Huang or Dex significantly decreased the levels of serum IgE in OVA mice. The BALF cells were collected 24 h after the OVA treatment. The different cell types were enumerated. *n*=10 in each group. *P<0.05, compared with the mice in the control group and ^#^*P*<0.05, compared with the mice in the OVA group.

To type and quantitate the infiltrated inflammatory cells more accurately, cells in the BALF were counted. Compared with levels in control mice, the amount of migrated inflammatory cells (EOSs, lymphocytes (LYMs), NEUs, and MACs) in the BALF from the OVA mice was significantly increased. However, administration of Huai Qi Huang or Dex significantly reduced the amount of migrated inflammatory cells (EOSs, LYMs, NEUs, and MACs) in the BALF from the OVA mice (Figure 2A–D).

### Huai Qi Huang decreased IgE in the serum

Elevated IgE in the serum is reported to be strongly associated with an increase in asthma severity [17]. The level of serum IgE was found to be significantly increased in the OVA mice as compared with the control mice. Administration of Huai Qi Huang or Dex significantly decreased the levels of serum IgE. This result indicated that Huai Qi Huang could lessen the severity of asthma (Figure 2E).

### Huai Qi Huang affected the levels of cytokines in the BALF

We then found that the level of IFN-γ, the principal Th1 cytokine, was significantly decreased in OVA mice as compared with the control mice. OVA mice also developed an inflammatory Th2 response in the lung with significantly elevated inflammatory Th2 cytokines (IL-4, IL-5, and IL-13) as compared with the control mice. The level of TNF-α was significantly increased in OVA mice as compared with the control mice. Additionally, the cardinal Th17 cytokine, IL-17, and the Treg-induced cytokine, IL-10 and TGF-β1, were also significantly increased in OVA mice. These results indicated the imbalance of Th1/Th2 and Treg/Th17 in OVA mice.

**Figure 3:**
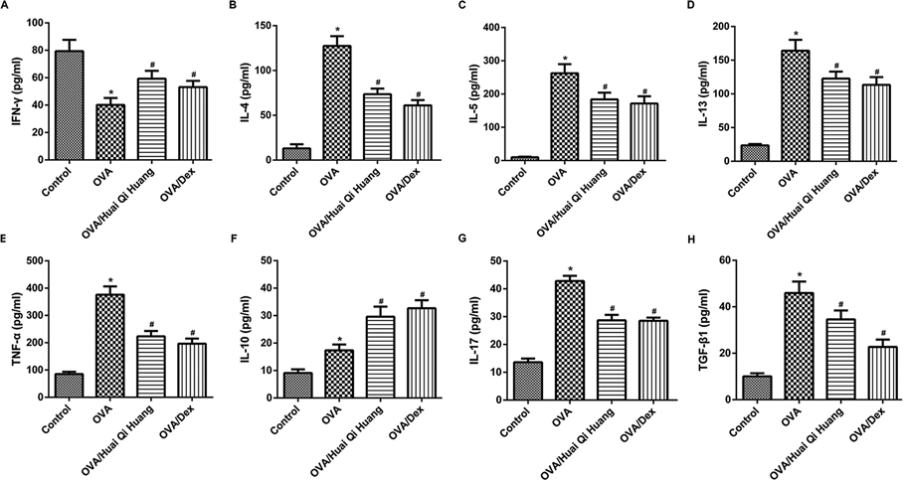
Huai Qi Huang affects the levels of cytokines in the BALF The BALF supernatants were collected 24 h after the OVA treatment. Th1 cytokines (IFN-γ (**A**), TNF-α (**E**)), Th2 cytokines (IL-4 (**B**), IL-5 (**C**), IL-13 (**D**)), a Th17 cytokine (IL-17 (**G**)), and Treg cytokines (IL-10 (**F**), TGF-β1(**H**)) were measured using ELISA kits. *n*=10 in each group. *P<0.05, compared with the mice in the control group and ^#^*P*<0.05, compared with the mice in the OVA group.

Huai Qi Huang or Dex treatment markedly increased the classic Th1 cytokine IFN-γ, but decreased inflammatory Th2 cytokines IL-4, IL-5, IL-13, and TNF-α in the OVA mice. Huai Qi Huang or Dex treatment also up-regulated the Treg cytokine IL-10 and down-regulated the Th17 cytokine IL-17 and decreased the level of TGF-β1. These results indicated that Huai Qi Huang regulates both the Th1/Th2 balance and the Th17/Treg balance (Figure 3).

### Huai Qi Huang influenced CD4^+^ T-cell subsets in the lung

**Figure 4:**
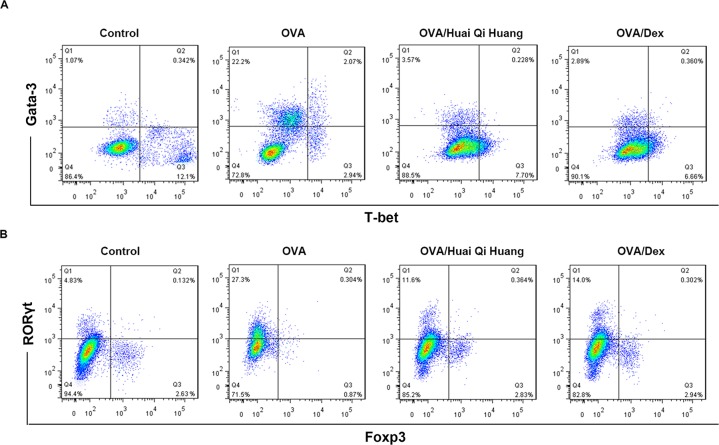
Huai Qi Huang influenced CD4^+^ T-cell subsets in the lung The lungs were collected 24 h after the OVA treatment and the single-cell suspension was prepared for flow cytometry. CD4^+^ cells were gated for analysis of the expression of T-bet and Gata-3 (**A**) or RORγt and Foxp3 (**B**). Representative result from ten independent experiments for each group was shown.

Furthermore, cell suspensions from the lung were stained with antibodies specific for CD4 and the master transcription factors for T-bet, Gata-3, RORγt, and Foxp3 to better understand the roles of CD4^+^ T subsets in the pathogenesis of asthma. OVA mice showed higher percentages of Gata-3^+^ cells (Th2 cells) and RORγt^+^ cells (Th17 cells), which was significantly decreased with the administration of Huai Qi Huang or Dex treatment. The percentages of T-bet^+^ cells (Th1 cells) and Foxp3^+^ cells (Treg cells) in the OVA mice were markedly decreased as compared with the control mice, but Huai Qi Huang or Dex treatment significantly altered these results (Figure 4). To sum up, our results suggested that Huai Qi Huang alleviated the imbalance of Th1/Th2 and Treg/Th17.

### Huai Qi Huang altered the mRNA expression levels of transcription factors for CD4^+^ T-cell subsets

**Figure 5:**
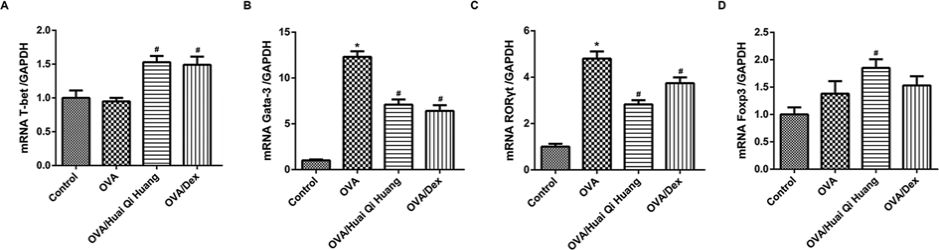
Huai Qi Huang altered the mRNA expression levels of transcription factors for CD4^+^ T-cell subsets The levels of transcription factors for T-bet (**A**), Gata-3 (**B**), RORγt (**C**), and Foxp3 (**D**) were examined with quantitative RT-PCR. The lungs were collected 24 h after the OVA treatment. The experiment was repeated at least six times. *n*=10 in each group. *P<0.05, compared with the mice in the control group and ^#^*P*<0.05, compared with the mice in the OVA group.

The levels of transcription factors for Th1, Th2, Th17, and Treg were examined with quantitative RT-PCR (Figure 5). The transcription of Th1 master transcription factor T-bet in control and OVA mice showed no significant differences. However, administration with Huai Qi Huang or Dex markedly increased the mRNA expression level of T-bet. In OVA mice, the markedly increased transcriptions of Gata-3 and RORγt were also found, and Huai Qi Huang or Dex treatment significantly decreased them. Besides, the transcription of Foxp3 in control and OVA mice showed no significant differences. However, administration with Huai Qi Huang markedly increased the mRNA expression level of Foxp3, whereas Dex treatment exerted no significant effects on the mRNA expression level of Foxp3. Therefore, Huai Qi Huang increased the ratio of not only T-bet/Gata-3 but also Foxp3/RORγt, regulating the balance of Th1/Th2 and Treg/Th17 in asthma.

## Discussion

In the present study, we first found that Huai Qi Huang inhibited infiltration of inflammatory cells into the lung, reduced influx of EOSs, LYMs, NEUs, and MACs in the BALF, and decreased IgE in the serum in OVA-induced asthma model mice. There is evidence for believing that asthma results from chronic airway inflammation involving a diversity of activated cells including mast cells, including EOSs, LYMs, NEUs, and MACs and epithelial cells [23]. These cells release proinflammatory cytokine mediators that augment and regulate airway inflammation, leading to airway hyperresponsiveness responsible for the chronic asthma symptoms of wheezing, dyspnea, and chest tightness [23]. Allergen-induced airway inflammation in asthma is associated with elevated IgE in blood plasma and involves infiltration of EOSs into the airway [24]. Thus, early intervention with anti-inflammatory agents that mitigate inflammatory changes may reverse airway obstruction and prevent progression of airway remodeling. Based on our results, Huai Qi Huang emerged as a potential anti-inflammatory agent for treatment of asthma.

Huai Qi Huang could also regulate Th1/Th2 and Treg/Th17 via the re-balance of cytokine profiles and ratios of transcription factors, T-bet/Gata-3 and Foxp3/RORγt in OVA-induced asthma model mice. Li et al. [20] showed that the levels of IL-4 and IL-17 were increased and the IFN-γ level was decreased in plasma and BALF, and the phagocytosis of alveolar MAC decreases in asthmatic rats. And Huai Qi Huang treatment increased IFN-γ expression in plasma and BALF and the phagocytosis of alveolar MAC in asthmatic rats. Wang et al. [25] suggested that Huai Qi Huang could relieve the hyperplasia of mesangial cells on immunity IgA nephritis model mouse, and the mechanism was closely related to up-regulating IFN-γ and IL-2 and down-regulating IL-4 secretion in splenic LYM and then correction of the imbalance of TH1/TH2.Liang et al. [26] indicated that Huai Qi Huang particles could reverse the immune imbalance of Th1 and Th2 in blood, and also linked to excessive regulation of Th17 cells, which affects IL-17 NEU-mediated inflammation and improves the cure rate of asthma. Kim et al. [27] suggested that oleanolic acid suppresses OVA-induced airway inflammation and Th2-mediated allergic asthma by modulating the transcription factors T-bet, GATA-3, RORγt, and Foxp3 transcription pathways. To some extent, these results were consistent with our investigations.

To sum up, our results showed that Huai Qi Huang could correct the imbalance of Th1/Th2 and Treg/Th17 in OVA-induced asthma model mice, indicating that this medicine may inhibit the development and severity of asthma.

## Abbreviations

BALF: bronchoalveolar lavage fluid
Dex: dexamethasone
EOS: eosinophil
Foxp3: forkhead box p3
Gata-3: Gata-binding protein 3
IFN: interferon
IL: interleukin
LYM: lymphocyte
MAC: macrophage
NEU: neutrophil
OVA: ovalbumin
RORγt: retinoic acid-related orphan receptor γt
Th: helper T
TNF: tumor necrosis factor
Treg: regulatory T cell
T-bet: T-box expressed in T cell
qRT-PCR: real time quantitative reverse transcription polymerase chain reaction
DEPC: diethy pyrocarbonate
GAPDH: glyceraldehyde-3-phosphate dehydrogenase
